# The Impact of Sex and 25(OH)D Deficiency on Metabolic Function in Mice

**DOI:** 10.3390/nu9090985

**Published:** 2017-09-07

**Authors:** Ryan J. Giblin, Ellen J. Bennett, Graeme R. Zosky, Renée M. Dwyer

**Affiliations:** School of Medicine, Faculty of Health, University of Tasmania, Hobart, Tasmania 7000, Australia; ryan.giblin@helsinki.fi (R.J.G.); ellen.bennett@utas.edu.au (E.J.B.); Graeme.zosky@utas.edu.au (G.R.Z.)

**Keywords:** sex, NAFLD, insulin resistance, vitamin D, 25(OH)D, type II diabetes

## Abstract

Both dietary fat and vitamin D deficiency have been linked with increased incidence of non-alcoholic fatty liver disease and insulin resistance. While sex differences in disease prevalence and severity are well known, the impact on disease pathogenesis remains unclear. To further explore the effect of these exposures on metabolic function, C57BL/6 male and female mice were weaned onto one of four diets; low fat vitamin D replete, low fat vitamin D deficient, or two high fat diets, one vitamin D replete and one deficient. Visceral fat, hepatic adiposity, and insulin resistance were measured after five and a half weeks. Vitamin D deficiency, independent of dietary fat, increased hepatic fat accumulation in both sexes (*p* = 0.003), although did not increase hepatic expression of interleukin-6 (*p* = 0.92) or tumor necrosis factor-α (*p* = 0.78). Males were observed to have greater insulin resistance (glucose area under the curve: *p* < 0.001, homeostatic model assessment for insulin resistance: *p* = 0.046), and have greater visceral adiposity (*p* < 0.001), while female mice had greater hepatic fat accumulation (*p* < 0.001). This study is the first to demonstrate vitamin D deficiency alone can cause hepatic accumulation while also being the first to observe higher liver fat percentages in female mice.

## 1. Introduction

Metabolic syndrome is a major health concern, with disease prevalence growing across the world [1]. With this metabolic dysfunction comes an increased risk of a wide range of related diseases, such as non-alcoholic fatty liver disease (NAFLD), insulin resistance, and type II diabetes [2,3]. Non-alcoholic fatty liver disease is a spectrum of diseases that affects around 25% of western populations. It can arise as simple fat accumulation in the liver, or progress through to non-alcoholic steatohepatitis, liver fibrosis, and finally liver cirrhosis [4]. Insulin resistance and type II diabetes are characterized by impaired insulin sensitivity and later in the disease, impaired insulin secretion [5].

Interestingly, there is evidence for sex differences in the prevalence and risk profile of these metabolic conditions. Epidemiological studies have shown that males have a higher risk of NAFLD [6,7], insulin resistance [8], type II diabetes [9], and metabolic syndrome [10] than females, whereas females tend to have more severe disease [9,11]. Despite these disparities between sexes, few experimental studies have directly assessed the impact of sex on metabolic dysfunction and disease progression [12,13].

While NAFLD, insulin resistance, and metabolic syndrome are connected in their prevalence and comorbidity, there remain questions as to what may be the driving factors connecting them. One possibility is vitamin D deficiency, which is widespread in developed countries [14] with prevalence increasing concomitantly with metabolic dysfunction [15]. Several association studies have linked vitamin D deficiency with metabolic syndrome [15], type II diabetes [16], insulin resistance [17], and NAFLD [18,19]. However, these associations are potentially confounded by the impact of chronic disease on physical activity levels which may limit sun exposure and, as a consequence, vitamin D synthesis [20].

Some experimental studies have addressed this issue. For example, a study by Roth and colleagues [21] found that vitamin D deficiency in combination with a western diet, but not alone, increased NAFLD and insulin resistance in male Sprague Dawley rats. Interestingly, vitamin D deficiency alone was sufficient to decrease insulin sensitivity. Geldenhuys et al. [22] found a similar effect in male mice whereby low serum vitamin D concentrations, in combination with a high fat diet, increased markers of NAFLD. Although, in contrast to the previous study, there was no evidence of vitamin D deficiency induced insulin resistance. As such, the impact of vitamin D deficiency on metabolic dysfunction requires further clarification. As highlighted earlier, both studies focused on male animals so the effect of sex on these responses is unclear.

This study aimed to assess the impact of vitamin D deficiency, alone or in combination with a high fat diet, on markers of metabolic function including visceral adiposity, hepatic lipid accumulation, and insulin sensitivity. We also compared these parameters between male and female mice to assess if sex influences the response to vitamin D deficiency and/or a high fat diet.

## 2. Materials and Methods

**Figure 1:**
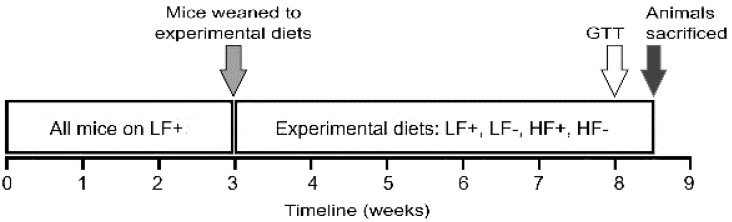
Experimental overview: Mice were weaned at three weeks of age separated by sex and assigned to one of the four experimental diets; low fat vitamin D replete (LF+), low fat vitamin D deficient (LF−), high fat vitamin D replete (HF+), or high fat vitamin D deficient (HF−). At eight weeks of age a glucose tolerance test (GTT) was performed, after three days’ recovery animals were sacrificed and tissue was harvested and stored for further analysis.

All experiments were performed according to the ethical guidelines of the National Health and Medical Research Council of Australia and with ethics approval from the University of Tasmania Animal Ethics Committee (Ethics No. A0015359). C57BL/6 mice were purchased from Jackson Laboratories (Bar Harbor, ME, USA) and housed at the Cambridge Animal Facility (University of Tasmania, Hobart, Australia). Prior to weaning, all mice, and their mothers, were kept on a low fat vitamin D replete diet (described below). After weaning, the mice were split into one of four experimental diets (described below) and maintained on these diets for five and a half weeks. Mice were separated by diet and sex and housed in a temperature controlled environment with a 12 h light/dark cycle and ad libitum access to water and the assigned diet. At eight weeks of age, following an overnight fast, a glucose tolerance test was performed. After three days’ recovery animals were overnight fasted a second time before being euthanized by interperitoneal injection of sodium pentobarbital (60 mg/kg bodyweight). At this time blood was taken for insulin and 25-hydroxy vitamin D (25(OH)D) measurements and tissue was harvested. An overview of the experimental protocol is shown in Figure 1.

### 2.1. Dietary Intervention

At weaning, mice were assigned to one of four experimental diets, identical to those previously described by Geldenhuys et al. [22] (Table 1). These included a low fat (5% wt/wt fat) feed containing either 2280 IU of vitamin D3/kg (LF+), or no added vitamin D3 (LF−), and a high fat (23.6% wt/wt fat) feed that also either contained 2280 IU of vitamin D3/kg (HF+), or no added vitamin D3 (HF−) (Specialty Feeds; Glen Forrest, Western Australia, Australia). The vitamin D deficient feeds were supplemented with 2% calcium, while the vitamin D replete feeds were supplemented with 1% calcium to ensure normocalcemia [23].

### 2.2. Serum Vitamin D (25(OH)D) Concentration

Vitamin D status was measured using a 25-Hydroxy Vitamin D EIA kit (Immunodiagnostic Systems, Tyne and Wear, UK) according to the manufacturer’s instructions.

### 2.3. Body Composition Changes

The effects of diet on somatic growth and body composition were assessed by measuring body weight, body length (snout-vent), and gonadal fat pad mass, as a marker of adiposity.

### 2.4. Histopathological Assessment of Liver Pathology

Non-alcoholic fatty liver disease was assessed (under blinded conditions) by quantifying the percentage of fat histologically using a stereological approach according to isotropic-uniform-random (IUR) sampling principles. The left and caudate liver lobes were fixed, embedded at random orientation, and then sectioned and stained with hematoxylin and eosin. Sectioning commenced at a random starting point between 0–1000 μm and 5 μm sections were taken every 1000 μm thereafter.

Liver sections were randomly selected and analyzed using Visiopharm Integrator System newCAST software (version 4.5.6, Visiopharm, Hørsholm, Denmark). The number of tissue point counts and fat point counts in each section were used to calculate the proportion of fat per liver using the stereology based volume density calculation method [24]. This approach has been found to be the most accurate and reliable way of quantifying liver fat [25].

### 2.5. Hepatic Cytokine Expression

The expression of hepatic inflammatory markers were assessed by enzyme-linked immunosorbent assay (ELISA). Protein from 22–30 mg of the median lobe of liver was extracted using a T-Per^®^ Tissue Protein Extraction Kit (Thermo Fisher Scientific, Scoresby, Victoria, Australia) with the addition of Halt™ Protease and Phosphatase Inhibitor Cocktail (Thermo Fisher Scientific, Scoresby, Victoria, Australia). Levels of tumor necrosis factor-α (TNF-α) and interleukin-6 (IL-6) were assessed by ELISA (DuoSet^®^, R&D Systems, Minneapolis, MN, USA), according to manufacturer’s instructions. Cytokine protein levels were normalized to total tissue protein concentration, assessed by Bradford assay (Commassie protein assay reagent, Sigma-Aldrich, Castle Hill, Australia).

### 2.6. Glucose Tolerance Test

The presence and severity of insulin resistance were assessed by glucose tolerance test (GTT). After five weeks on the assigned diet, mice were fasted overnight for at least 16 h. Blood was obtained via tail snip and blood glucose measured using an Accu-Chek Performa glucometer (Roche, Bella Vista, NSW, Australia). Fasting blood glucose concentrations were recorded before mice were administered a glucose challenge (1 g/kg body weight) via an intraperitoneal injection. Blood glucose concentration was then assessed every 15 min for 90 min post-injection.

### 2.7. Serum Insulin Concentration

Fasting insulin concentration was measured using a mouse insulin ELISA kit (Mercodia, Uppsala, Sweden), according to the manufacturer’s instructions.

### 2.8. Statistical Analyses

Differences between groups were tested using two-way ANOVA with Holm-Sidak post-hoc tests (SigmaPlot 12.5; SyStat Software Inc., San Jose, CA, USA). Multiple linear regression was used to assess the associations between continuous variables (Stata 14 statistical software package, Stata Corp, College Station, TX, USA). Data were transformed to satisfy the assumptions of normality and equal variance where appropriate. *p* < 0.05 was considered statistically significant. Data are presented as mean ± standard deviation, unless explicitly stated otherwise.

## 3. Results

### 3.1. Serum 25-Hydroxy Vitamin D Concentration

**Figure 2:**
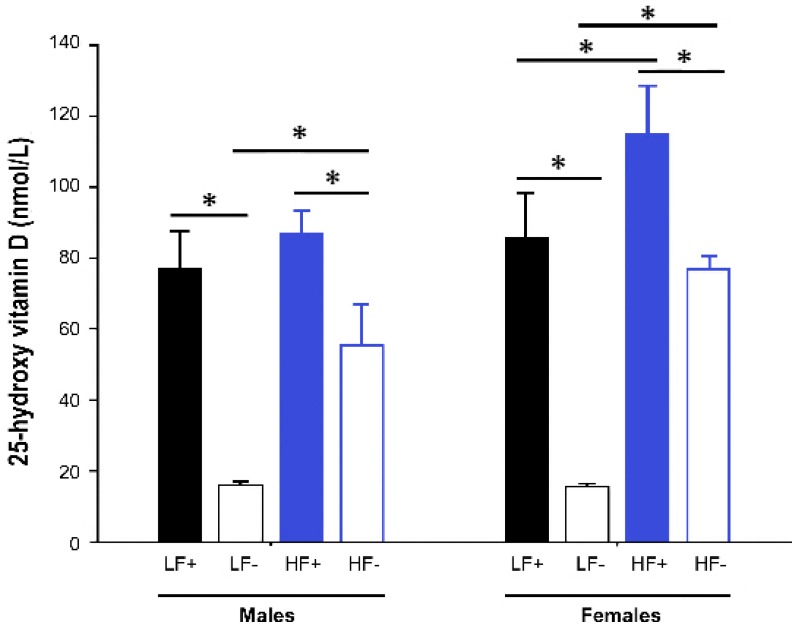
Serum 25-hydroxy vitamin D concentrations of adult C57BL/6 mice fed a low fat vitamin D replete (LF+), low fat vitamin D deficient (LF−), high fat vitamin D replete (HF+), and high fat vitamin D deficient (HF−) diet. Data shown as mean ± SD with *n* = 7–8 for each group, * *p* < 0.05.

Both male and female mice fed vitamin D deficient diets had significantly lower 25(OH)D concentrations than their respective vitamin D replete controls (male LF− vs. LF+: *p* < 0.001; male HF− vs. HF+: *p* < 0.001; female LF− vs. LF+: *p* < 0.001; female HF− vs. HF+: *p* < 0.001). However, the mice on the high fat vitamin D deficient diet had higher levels of 25(OH)D than the deficient mice on the low fat diet (male HF− vs. LF-: *p* < 0.001; female HF− vs. LF−: *p* < 0.001), indicating that the high fat diet feed increased 25(OH)D concentrations (Figure 2).

As a result, the mice on the HF− diet were not vitamin D deficient (25(OH)D < 50 nmol/L). Consequently, data were analyzed, with sex as a factor in each, in a hierarchical fashion as follows:

- The effect of a high fat diet was assessed by comparing outcomes in the replete mice on high fat and low fat diets (LF+ vs. HF+)
- The effect of vitamin D deficiency was assessed by comparing outcomes in the low fat groups (LF+ vs. LF−)
- The modifying effect of vitamin D concentration on the high fat response was assessed by examining the relationship between vitamin D levels (as a continuous variable) and outcomes in mice on the high fat diets (both HF+ and HF−).

### 3.2. Body Weight and Length

**Figure 3:**
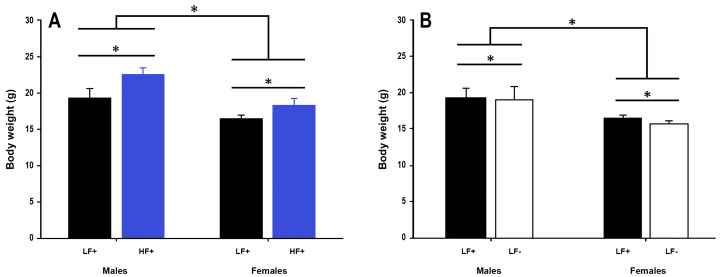
Body weight of adult C57BL/6 mice fed a low fat vitamin D replete (LF+), low fat vitamin D deficient (LF−), and high fat vitamin D replete (HF+) diet. (**Panel A**) shows the effect of dietary fat within vitamin D replete mice; (**Panel B**) shows the effect of vitamin D deficiency within low fat diet fed mice. Data shown as mean ± SD, male groups *n* = 9, female groups *n* = 7–8, * *p* < 0.05.

A high fat diet led to increased body weight in both sexes compared to a low fat control diet (*p* < 0.001, Figure 3A).

Vitamin D deficiency decreased body weight in both male and female mice (*p* = 0.04, Figure 3B), although the magnitude of this effect was small, ~0.6 g (3.3% total body weight). 25(OH)D concentration did not significantly modify the effect a high fat diet had on body weight (*p* = 0.62, Table 2). Males were longer than females (*p* < 0.001), and a high fat diet led to longer bodies in both sexes (*p* = 0.02), although the magnitude of the diet effect was small, ~1.5 mm (1.9% total length) (Supplementary Figure S1).

### 3.3. Gonadal Fat Pad Weight

**Figure 4:**
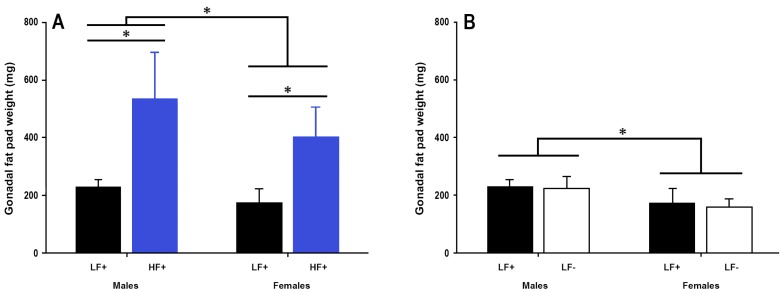
Gonadal fat pad weight of adult C57BL/6 mice fed a low fat vitamin D replete (LF+), low fat vitamin D deficient (LF−), and high fat vitamin D replete (HF+) diet. (**Panel A**) shows the effect of dietary fat within vitamin D replete mice; (**Panel B**) shows the effect of vitamin D deficiency within low fat diet fed mice. Data shown as mean ± SD, male groups *n* = 9, female groups *n* = 7–8, * *p* < 0.05.

A high fat diet increased fat pad weight in both sexes (*p* < 0.001, Figure 4A). There was no effect of vitamin D deficiency on gonadal fat pad weight in either sex (*p* = 0.39, Figure 4B), however 25(OH)D concentration was significantly related to fat pad weight in mice fed a high fat diet (*p* = 0.049, Table 2) such that higher 25(OH)D levels were associated with greater fat pad weight.

### 3.4. Hepatic Fat Content

**Figure 5:**
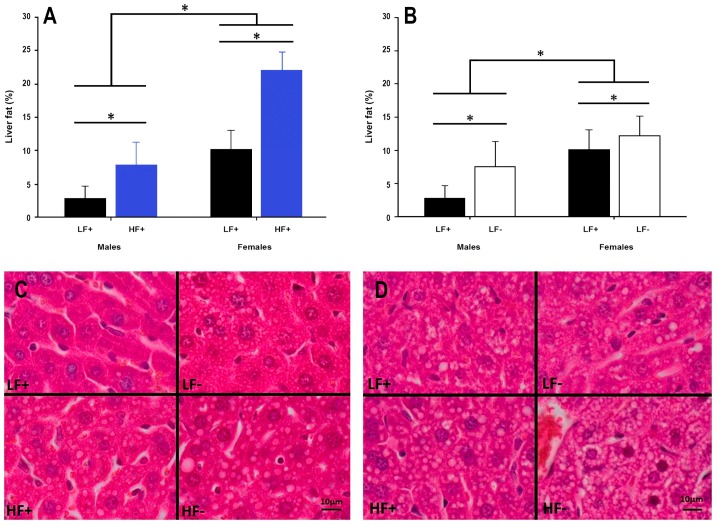
Hepatic fat content in adult C57BL/6 mice fed a low fat vitamin D replete (LF+), low fat vitamin D deficient (LF−), high fat vitamin D replete (HF+), and high fat vitamin D deficient (HF−) diet. (**Panel A**) shows the effect of dietary fat within vitamin D replete mice; (**Panel B**) shows the effect of vitamin D deficiency within low fat diet fed mice. Data shown as mean ± SD, male groups *n* = 9, female groups *n* = 7–8, * *p* < 0.05; (**Panel C**) shows representative histological microscope images of liver fat content in male mice; (**Panel D**) shows the equivalent images in female mice. Both high fat and vitamin D deficient diets caused increased lipid accumulation in both sexes, although females had greater lipid accumulation across each comparison.

A high fat diet increased hepatic fat content in both sexes (*p* < 0.001, Figure 5A,C,D). Interestingly, vitamin D deficiency alone increased liver fat accumulation in both male and female mice (*p* = 0.003, Figure 5B–D), while decreasing 25(OH)D concentrations were also associated with increased liver fat percentage in mice on a high fat diet (*p* = 0.006, Table 2).

### 3.5. Hepatic Inflammation

**Figure 6:**
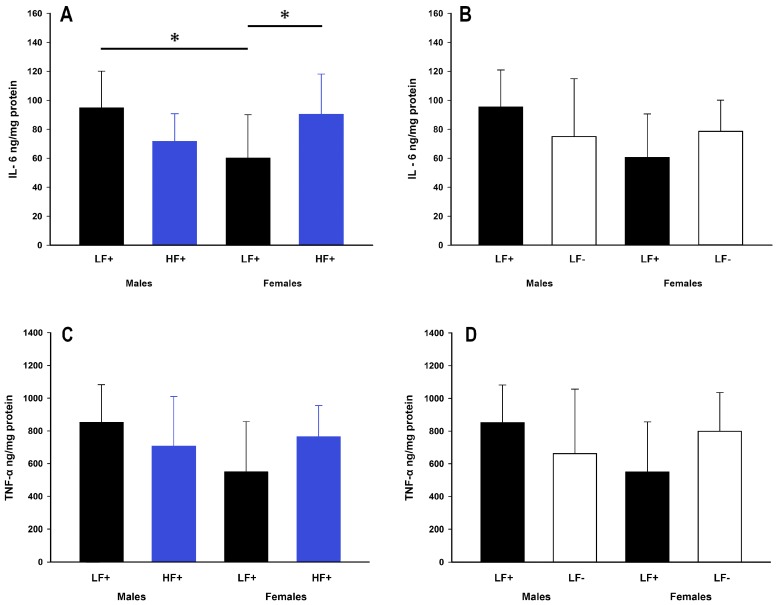
Hepatic inflammatory cytokine expression of adult C57BL/6 mice fed a low fat vitamin D replete (LF+), low fat vitamin D deficient (LF−), and high fat vitamin D replete (HF+) diet. (**Panel A**) shows the effect of dietary fat within vitamin D replete mice on IL-6 protein expression; (**Panel B**) shows the effect of vitamin D deficiency within low fat diet fed mice on IL-6 protein expression; (**Panel C and D**) show the effect of dietary fat and vitamin D deficiency respectively on TNF-α protein expression. Data shown as mean ± SD, male groups *n* = 9, female groups *n* = 7–8, * *p* < 0.05.

While a high fat diet did not impact expression of IL-6 (*p* = 0.50) or TNF-α (*p* = 0.50), there were some within group differences. Within low fat replete fed mice, males had higher expression of IL-6 compared to females (male LF+ vs. female LF+: *p* = 0.01, Figure 6A). Within females, but not males (*p* = 0.06), a high fat diet caused increased IL-6 expression compared to a low fat diet (female LF+ vs. female HF+: *p* = 0.03, Figure 6A). There was no difference in the expression of TNF-α across all low and high fat groups (*p* > 0.056 for all comparisons, Figure 6C). Similarly, vitamin D levels were not significantly related to IL-6 (*p* = 0.06) or TNF-α (*p* = 0.20) expression in mice on a high fat diet (Table 2).

### 3.6. Insulin Sensitivity

**Figure 7:**
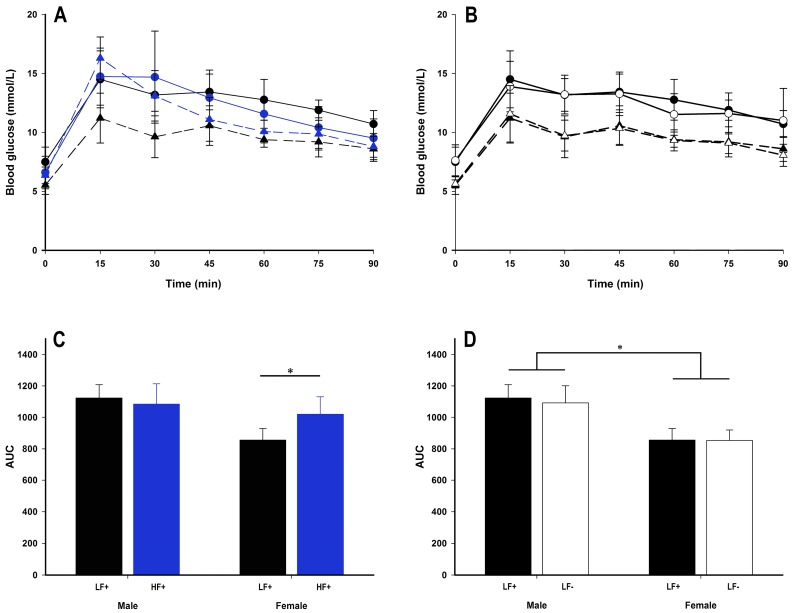
Glucose tolerance test (GTT) data of adult C57BL/6 mice fed a low fat vitamin D replete (LF+; black symbols), low fat vitamin D deficient (LF−; white symbols), and high fat vitamin D replete (HF+; blue symbols) diet. Panel A and B show the GTT time course (males represented as circles with solid lines, females as triangles with dotted lines); panel C and D show the effect of dietary fat and vitamin D deficiency respectively on total area under the curve (AUC) calculated from the GTT time course. Data shown as mean ± SD, male groups *n* = 9, female groups *n* = 7–8, * *p* < 0.05.

A high fat diet had some impact on glucose tolerance in female, but not male mice (Figure 7A). While there were no differences in fasting blood glucose, fasting insulin, or homeostatic model assessment for insulin resistance (HOMA-IR) (*p* > 0.057 for all comparisons, Table 3), within females, a high fat diet increased area under the curve (AUC) (female LF+ vs. HF+: *p* = 0.006, Figure 7C) and GTT peak concentration (female LF+ 11.93 ± 1.34 mmol/L, female HF+ 16.30 ± 1.79 mmol/L: *p* = 0.001, Supplementary Figure S2A). Compared to female mice, males had higher AUC (male vs. female within LF: *p* < 0.001, Figure 7D; within HF: *p* = 0.02, Table 2), GTT peak (male 15.08 ± 1.52 mmol/L vs. female 12.05 ± 1.60 mmol/L within LF: *p* < 0.001, Supplementary Figure S2B), HOMA-IR (male vs. female within LF: *p* = 0.046, Table 4 within HF: *p* = 0.04, Table 2), and within a high fat diet at least, fasting insulin (*p* = 0.004, Table 2) indicating greater insulin resistance.

Vitamin D deficiency did not alter any insulin resistance measure (*p* > 0.087 for all comparisons, Figure 7B,D, Table 2 and Table 4) for either sex. However, increased 25(OH)D concentrations were associated with increased fasting insulin (*p* = 0.007) and HOMA-IR (*p* = 0.04) for mice on a high fat diet (Table 2).

## 4. Discussion

The current study showed that a high fat diet causes increased fat deposition in both the visceral fat pads and liver. Interestingly, vitamin D deficiency alone caused a similar increase in fat accumulation within the liver, while also causing a mild decrease in insulin sensitivity. This is the first study to demonstrate that vitamin D deficiency alone can cause increased hepatic fat accumulation, substantiating the relationship between vitamin D deficiency and increased NAFLD seen in human studies [18,19]. Importantly, this study also showed evidence for metabolic differences between male and female mice. Male mice had increased visceral adiposity and decreased insulin sensitivity. This is consistent with evidence from human studies, showing that males are more susceptible to visceral fat accumulation [26] and insulin resistance [8]. In contrast, female mice had greater fat deposits in the liver across all diets, and diminished insulin sensitivity on the high fat diet. The current study provides novel evidence that females may be more susceptible to hepatic fat accumulation than males.

Importantly, the five and a half week high fat dietary intervention used in this study was of sufficient duration to observe a redistribution in body weight and visceral adiposity. Similarly, striking differences in hepatic fat content, the key marker of NAFLD, were observed between diets whereby mice fed a high fat diet had increased liver fat percentage compared to mice fed a low fat diet. This finding is consistent with previous studies in rats [21], and C57BL/6 mice [22], which have used a more prolonged dietary intervention (>10 weeks) to induce an insulin resistant state. Therefore, the results from the current studies shorter five and a half week dietary intervention assess an earlier time-point in the development of metabolic dysfunction and show that hepatic fat accumulation and alterations in fat storage occur during the early stages of disease progression.

Additionally, mice on a low fat vitamin D deficient diet had greater liver fat percentage than low fat vitamin D replete mice, and in mice on a high fat diet, greater liver fat percentage was associated with decreasing serum 25(OH)D concentrations. Both findings suggest that vitamin D is a key nutrient in hepatic lipid homeostasis, with decreased vitamin D leading to increased fat accumulation in the liver. Previous studies have shown vitamin D deficiency in combination with a high fat diet increases liver fat in both rats [21] and mice [22], however this study is the first to demonstrate that vitamin D deficiency alone can cause increased hepatic fat content and is also the first to demonstrate that sex can influence the extent of this accumulation. The current results suggest that the association between vitamin D deficiency and increased NAFLD in humans [18] could be causal, with 25(OH)D deficient individuals potentially at risk of developing NAFLD. While several randomized controlled trails [27,28] have shown vitamin D supplementation has not reversed NAFLD state in humans, these trials have focused on subjects with established disease. As such, these studies are unable to assess if there is any causal link between vitamin D deficiency and the risk of developing disease or its impact on disease progression. Furthermore, these trials primarily assess liver enzymes as markers of NAFLD and therefore have not directly assessed hepatic fat content. Our data suggest it may be important for further trials to investigate if vitamin D supplementation can prevent or limit liver fat deposition during early disease progression.

Intriguingly, female mice had higher liver fat percentages than male mice, across all group comparisons. This is inconsistent with the results of a previous study [29] examining potential sex differences in liver fat accumulation of C57BL/6 mice fed a high fat diet. One potential explanation for the inconsistent results is the method used to quantify fat in the liver as many animal studies have used semi-quantitative methods [21,29] to determine hepatic fat percentage. A strength of the current study is the use of stereological point counting, which is more sensitive at quantifying differences, and is the preferred method for liver fat assessment [25].

It is recognized that inflammatory cytokines play a key role in the progression of NAFLD, from passive fat accumulation to steatohepatitis [30]. Increased cytokine expression, particularly IL-6 and TNF-α, is a proposed mechanism for vitamin D deficient induced progression of NAFLD that has been demonstrated in both humans [31] and animal models [21]. There was however, nothing to suggest that IL-6 or TNF-α had increased in concert with hepatic fat accumulation in our study. This may be due to the short intervention protocol which has allowed early events in the metabolic disturbance to be assessed. Inflammation is likely to occur over a more prolonged period and possibly as a result of the increased hepatic fat accumulation rather than being a causative factor. Our data suggests that vitamin D deficiency increases hepatic fat accumulation independent of common hepatic inflammation mediators.

Male mice appeared to be less insulin sensitive than female mice. Specifically, compared to female mice, males had increased AUC, GTT peak, fasting insulin, and HOMA-IR scores. However, female mice were affected by a high fat diet with increased blood glucose concentrations in the first 30 min following the glucose challenge. This led to increased AUC and GTT peak compared to low fat diet fed females. Interestingly, male mice were not adversely affected by a high fat diet. This suggests that although male mice were less insulin sensitive than females at baseline, females are more susceptible to the subsequent effects of exposure to a high fat diet. This may explain the sex discrepancy in the human data whereby males are at higher risk of disease [8] (higher baseline insulin resistance) while females often have more severe disease (more sensitive to metabolic insults) [11].

While there were significant associations between increasing 25(OH)D and both fasting insulin concentrations and HOMA-IR scores in mice on a high fat diet, the size of the effect was modest. Furthermore, there were no associations between 25(OH)D and fasting blood glucose, AUC, or GTT peak. Equally, mice on a low fat vitamin D deficient diet had no alterations in insulin sensitivity compared to vitamin D replete mice. As the lard component of the high fat diet was the most likely source of erroneous vitamin D in the HF− group, there is a possibility that the within high fat diet vitamin D associations found in the current study may be at least partly related to the amount of high fat diet consumed. Animals that ate more, would have not only consumed higher fat content, but also higher amounts of vitamin D, thus resulting in the associations between higher 25(OH)D and plasma insulin and HOMA-IR. This suggests that 25(OH)D concentration has a minimal effect on insulin sensitivity. Still, it must be stated that the association between 25(OH)D and increased fasting plasma insulin coupled with the association between 25(OH)D and decreased liver fat accumulation seen within the high fat diet fed animals is highly unusual for this model. Of course it is possible that the model used was of insufficient duration to observe the true or longer term effects, which is plausible given that insulin sensitivity was only marginally diminished in many animals. In future studies it may be appropriate to use a longer dietary intervention to allow greater insulin resistance to develop, in order to tease out the full effect on insulin sensitivity caused by 25(OH)D deficiency.

There are a number of limitations that should be acknowledged in the current study. The high fat vitamin D deficient diet seemingly contained unintended vitamin D, leaving mice on this diet with significantly higher serum 25(OH)D concentrations than low fat vitamin D deficient fed mice. This most likely occurred as a result of the animal fat (lard) component of the commercially manufactured high fat diet, containing inadvertent fat-soluble vitamin D. As such our study did not include a true high fat vitamin D deficient group, leading to our stratified analytical approach. Additionally, previous studies [21,22] have used extended intervention periods, that potentially allowed more pronounced insulin resistance to develop and consequently highlighted biological differences more starkly. While the current study aimed to uncover early disease progression, it accordingly resulted in only modest disturbances to insulin sensitivity. Interestingly, while only slight decreases to insulin sensitivity were observed, large differences were detected in hepatic fat content between groups. This could have implications for early detection of metabolic dysfunction. It should be noted that food intake and physical activity were not measured in the current study. Given the lack of difference in gonadal fat pad weight and minimal body weight differences seen within low fat diet fed animals, major differences to physical activity and food intake appear unlikely. Nevertheless, these factors could partially explain some of the other metabolic differences observed within low fat diet groups.

While acknowledging these limitations, the current study has enhanced our understanding of the potential impact of vitamin D deficiency on the development of early metabolic dysfunction. The majority of the metabolic disturbances were due to a high fat diet, however vitamin D deficiency had a significant effect on hepatic fat accumulation regardless of diet composition. This suggests the relationship between vitamin D deficiency and NAFLD observed in human populations [18,19] could be causal. Major sex differences were uncovered in the current study. Consistent with the human data, the current study found female mice were generally more insulin sensitive [8], weighed less, and had less visceral fat mass [26] than male mice. Contrary to previous findings in humans [6,7], female mice also had greater hepatic fat deposition than male mice, although this may be due to methodological differences. Importantly, this study shows that hepatic fat accumulation may be one of the earliest signs of metabolic dysfunction, with insulin resistance taking longer to fully develop.

## 5. Conclusions

Overall, this study suggests that 25(OH)D deficiency and a high fat diet have independent adverse effects on metabolic health and show that vitamin D deficiency alone, may contribute to an increase in hepatic fat content. This data shows that hepatic fat accumulation may be an early event in the progression of metabolic dysfunction. Our data also suggests that males are at greater risk of developing increased visceral adiposity and insulin resistance while females are at greater risk of hepatic fat accumulation, demonstrating the need to study both sexes to fully understand the mechanisms that contribute to disease development and its progression.

## Figures and Tables

**Table 1:** Nutrient composition of low and high fat diets.

Diet	Carbohydrate			Protein	Fat		Micronutrients *
	Sucrose	Starch	Fiber	Casein	Canola Oil	Lard	
Low Fat	10.0	53.4	5.0	20.0	5.0	-	6.6
High Fat	10.0	30.6	5.0	20.0	2.9	20.7	10.8

Data presented as percentage by weight. * e.g., calcium, magnesium, potassium, trace minerals.

**Table 2:** Multiple linear regression analysis for variables relating to metabolic function of adult C57BL/6 mice fed either a high fat vitamin D replete (HF+) or high fat vitamin D deficient (HF−) diet.

Outcome	25-Hydroxy Vitamin D		Sex	
	β (95% CI)	*p*	β (95% CI)	*p*
Body weight	0.007 (−0.021 0.035)	0.62	5.7 (4.5 7.0)	**<0.001**
Body length	0.000 (−0.050 0.051)	0.99	5.1 (2.8 7.4)	**<0.001**
Gonadal fad pad	2.507 (0.012 5.002)	**0.049**	234.3 (120.9 347.6)	**<0.001**
Liver fat %	−0.162 (−0.272 −0.051)	**0.006**	−15.9 (−20.9 −10.9)	**<0.001**
GTT peak	0.021 (−0.029 0.071)	0.40	1.9 (−0.4 4.2)	0.10
AUC	−1.051 (−3.560 1.458)	0.40	134.7 (20.7 248.6)	**0.02**
Fasting glucose	0.030 (−0.007 0.068)	0.11	1.3 (−0.4 3.0)	0.12
Fasting insulin	0.013 (0.006 0.019)	**0.007**	0.6 (0.2 1.0)	**0.004**
HOMA-IR	0.009 (0.001 0.018)	**0.04**	0.4 (0.0 0.8)	**0.04**
IL-6 protein	0.199 (−0.520 0.919)	0.06	−32.7 (−65.6 0.2)	0.05
TNF-α protein	4.117 (−2.368 10.602)	0.20	−152.5 (−449.1 144.1)	0.30

Regression co-efficient (β), 95% confidence intervals (95% CI), and *p* values displayed for the continuous variable ‘25-hydroxy vitamin D’ and categorical variable ‘Sex’ (positive co-efficient favors males, negative favors females). GTT = glucose tolerance test, AUC = area under the curve, HOMA-IR = homeostatic model assessment for insulin resistance, IL-6 = interleukin-6, TNF-α = tumor necrosis factor-α. Male groups *n* = 9, female groups *n* = 7–8, **bold** numbers signifies *p* < 0.05.

**Table 3:** Insulin resistance measures for adult C57BL/6 mice fed either a low fat vitamin D replete (LF+) or high fat vitamin D replete (HF+) diet.

Sex	Diet	Fasting Blood Glucose	Fasting Insulin	HOMA-IR
		(mmol/L)	(mU/L)	
Males	LF+	10.6 ± 1.9	4.06 ± 0.74	1.9 ± 0.5
	HF+	10.6 ± 1.8	4.06 ± 0.54	1.9 ± 0.5
Females	LF+	8.9 ± 1.8	3.96 ± 0.79	1.5 ± 0.2
	HF+	9.7 ± 1.6	3.90 ± 0.57	1.7 ± 0.4

HOMA-IR = homeostatic model assessment for insulin resistance. Data shown as mean ± SD, male groups *n* = 9, female groups *n* = 7–8.

**Table 4:** Insulin resistance measures for adult C57BL/6 mice fed either a low fat vitamin D replete (LF+) or low fat vitamin D deficient (LF−) diet.

Sex	Diet	Fasting Blood Glucose	Fasting Insulin	HOMA-IR
		(mmol/L)	(mU/L)	
Males	LF+	10.6 ± 1.9	4.06 ± 0.74	1.9 ± 0.5 *
	LF−	9.8 ± 2.7	3.73 ± 0.41	1.6 ± 0.6 *
Females	LF+	8.9 ± 1.8	3.96 ± 0.79	1.5 ± 0.2
	LF−	8.4 ± 1.0	3.56 ± 0.29	1.4 ± 0.2

HOMA-IR = homeostatic model assessment for insulin resistance. Data shown as mean ± SD, male groups *n* = 9, female groups *n* = 7–8. * Significant difference between sexes (*p* = 0.046).

## Supplementary Materials

The following are available online at www.mdpi.com/2072-6643/9/9/985/s1, Figure S1: Body length, Figure S2: Peak blood glucose concentrations.
