# Supplementary material for: The Impact of Sex and 25(OH)D Deficiency on Metabolic Function in Mice
Source: Nutrients. 2017 Sep 7;9(9):985. doi: 10.3390/nu9090985 (PMC5622745; doi:10.3390/nu9090985)
Supplement: Supplementary file 1 [file nutrients-09-00985-s001.pdf]

Supplementary Materials: The Impact of Sex and 25(OH)D Deficiency on Metabolic Function in Mice

Ryan J. Giblin, Ellen J. Bennett, Graeme R. Zosky and Renée M. Dwyer

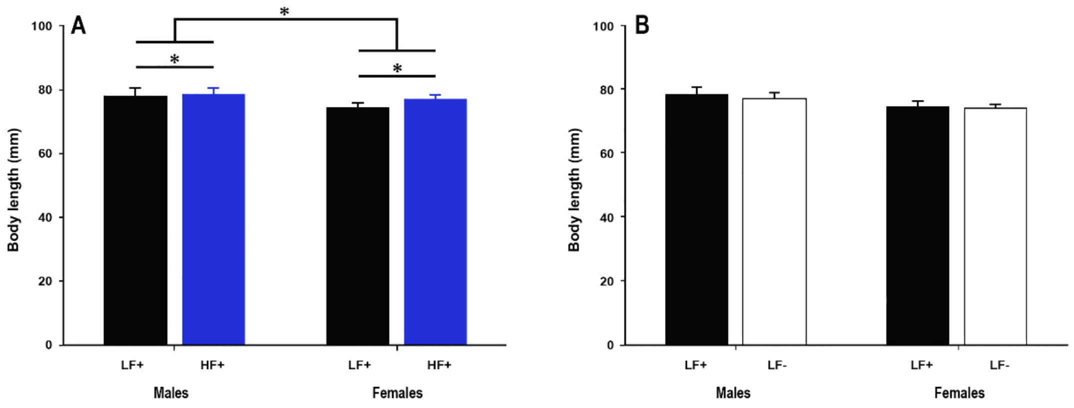

Figure S1. Body length.

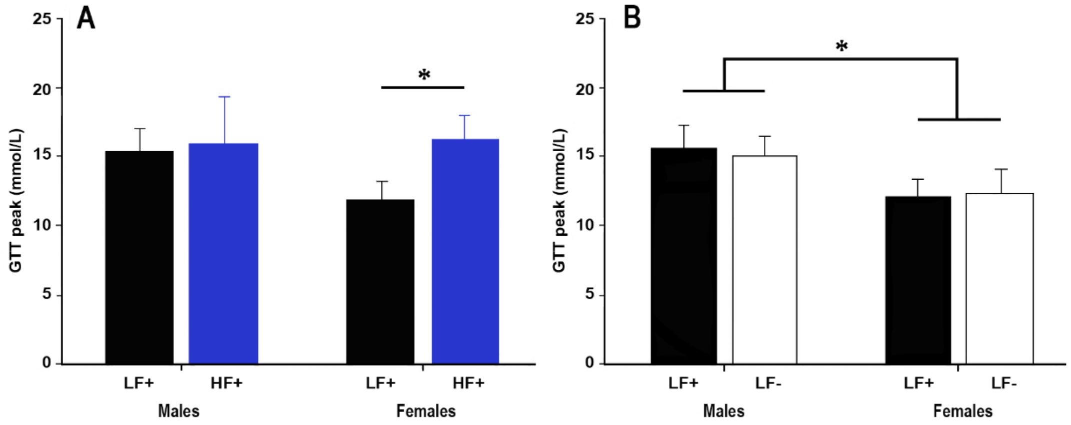

Figure S2. Peak blood glucose concentrations.
